# Effects of Chlorophenoxy Herbicides and Their Main Transformation Products on DNA Damage and Acetylcholinesterase Activity

**DOI:** 10.1155/2014/709036

**Published:** 2014-03-27

**Authors:** Sofia Benfeito, Tiago Silva, Jorge Garrido, Paula B. Andrade, M. J. Sottomayor, Fernanda Borges, E. Manuela Garrido

**Affiliations:** ^1^Departamento de Engenharia Química, Instituto Superior de Engenharia do Porto (ISEP), Instituto Politécnico Porto, 4200-072 Porto, Portugal; ^2^CIQ/Departamento de Química e Bioquímica, Faculdade de Ciências, Universidade do Porto, 4169-007 Porto, Portugal; ^3^REQUIMTE/Laboratório de Farmacognosia, Departamento de Química, Faculdade de Farmácia, Universidade do Porto, 4050-313 Porto, Portugal

## Abstract

Persistent pesticide transformation products (TPs) are increasingly being detected among different environmental compartments, including groundwater and surface water. However, there is no sufficient experimental data on their toxicological potential to assess the risk associated with TPs, even if their occurrence is known. In this study, the interaction of chlorophenoxy herbicides (MCPA, mecoprop, 2,4-D and dichlorprop) and their main transformation products with calf thymus DNA by UV-visible absorption spectroscopy has been assessed. Additionally, the toxicity of the chlorophenoxy herbicides and TPs was also assessed evaluating the inhibition of acetylcholinesterase activity. On the basis of the results found, it seems that AChE is not the main target of chlorophenoxy herbicides and their TPs. However, the results found showed that the transformation products displayed a higher inhibitory activity when compared with the parent herbicides. The results obtained in the DNA interaction studies showed, in general, a slight effect on the stability of the double helix. However, the data found for 4-chloro-2-methyl-6-nitrophenol suggest that this transformation product can interact with DNA through a noncovalent mode.

## 1. Introduction

World population is expected to grow by over a third between 2009 and 2050. The projections show that feeding a world population of 9.1 billion people in 2050 would require raising overall food production by about 70 percent between 2005/07 and 2050 [1]. The rate of growth in world demand for agricultural goods may lead to increased use of chemical control products since these substances can contribute considerably to increasing yields and improve farm revenues.

Pesticides, a broad group of biologically active compounds used for pest management, are among the most widely used chemicals in the world and also among the most dangerous to environmental and human health. The impact of pesticide molecules on the environment depends on several factors: their toxicity, their bioaccumulation and long-term effects, their transport between different compartments, and their persistence in the environment [2]. There is also increasing interest in their transformation products (TPs), since these by-products can play a significant role in defining the impact of pesticides on both human health and the natural ecosystems. The formation and environmental presence of TPs thus add further complexity to chemical risk assessment. TPs may contribute significantly to the risk posed by the parent compound (a) if they are formed with a high yield, (b) if they are more persistent or more mobile than the parent compounds, or (c) if they have higher toxicity [3].

The adverse health effects of pesticide use have long been established, with links to neurologic and endocrine (hormone) system disorders, birth defects, cancer, and other diseases [4, 5]. Although, in general, pesticide TPs show lower toxicity to biota than the parent compounds, in some cases TPs are more toxic and represent a greater risk to the environment and health than the parent molecules [3, 6]. Therefore, toxicological evaluation of pesticide TPs is considered to be an emerging issue.

Acetylcholinesterase enzyme (AChE) is very important for the central nerve system of humans and insects. AChE is an enzyme that degrades, through its hydrolytic activity, the neurotransmitter acetylcholine and is critically important for the regulation of neurotransmission at synapses in all areas of the nervous system [7]. If AChE activity is blocked, acetylcholine accumulates at cholinergic receptor sites, thereby excessively stimulating the cholinergic receptors. This can lead to various clinical complications including fibrillation, leading ultimately to death [7]. So, large-scale inactivation of AChE has lethal consequences for any organism with a nervous system. It is well demonstrated that AChE is inhibited by neurotoxins, namely, organophosphates and carbamate pesticides, and many other compounds [8, 9]. In fact, recent reports have also described that some fungicides and herbicides used in agrochemicals can also damage AChE [10, 11]. The evaluation of AchE inhibitory activity can help to determine the toxicity levels of a diversity of net pesticides and their degradation products.

Besides the enzymatic damage in the biologic systems, pesticides have been shown to have the capacity of interacting with the DNA. Chemicals that interact with DNA can cause direct damage by covalent modifications, such as adducts or strand breaks, or can perturb DNA and chromatin function by noncovalent binding [12–14]. Thus, the preliminary evaluation of DNA damage is considered to be of high significance for the study of the putative interactions of pesticides and their TPs with DNA and for the screening of their mutagenic properties.

Chlorophenoxy herbicides are used worldwide as plant growth regulators for agricultural and nonagricultural purposes [15, 16]. These herbicides can be easily transferred to surface and ground waters due to their polar nature and relatively good solubility which increase the risks of contamination and consequently the environmental damage [17]. Based on epidemiologic studies, phenoxy acid herbicides have been classified by the International Agency for Research on Cancer (IARC) as possibly carcinogenic to humans (category 2B) [18]. The most commonly reported transformation intermediates of phenoxy acid degradation are the corresponding chlorophenols and their nitroderivatives, the latter being environmentally more persistent than the parent molecules [19, 20]. In fact, the key metabolite in the degradation of phenoxy acids is the corresponding chlorophenol that results from cleavage of the ether bond in the parent compound. The occurrence of nitrophenols as transformation products is a consequence of a photochemical nitration process of the corresponding phenols [19]. Due to the environmental occurrence of these compounds, their risk assessment is very relevant because the nitration of chlorophenols reduces their acute toxicity but the nitroderivatives could have more marked long-term effects, associated with their genotoxicity [20, 21].

**Figure 1:**
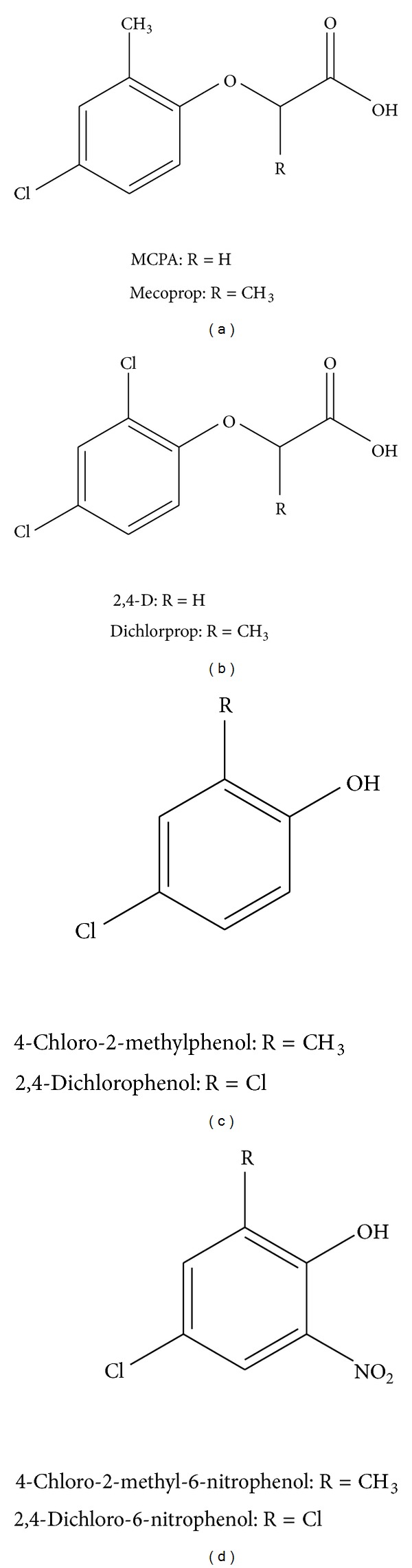
Chemical structures of the chlorophenoxy herbicides and their main environmental transformation products: (a) MCPA and mecoprop, (b) 2,4-D and dichlorprop, (c) 4-chloro-2-methylphenol and 2,4-dichlorophenol, and (d) 4-chloro-2-methyl-6-nitrophenol and 2,4-dichloro-6-nitrophenol.

The aim of this work was to evaluate the interaction of four chlorophenoxy herbicides (MCPA, mecoprop, 2,4-D, and dichlorprop) as well as their main transformation products (2,4-dichlorophenol, 4-chloro-2-methylphenol, 2,4-dichloro-6-nitrophenol, and 4-chloro-2-methyl-6-nitrophenol) with calf thymus DNA by UV-visible absorption spectroscopy (Figure 1). Thermodynamic parameters of DNA thermal denaturation, in solutions containing the herbicides and their TPs, have been evaluated to interpret the mode of interaction. Furthermore, the toxicity of the chlorophenoxy herbicides and TPs was also assessed evaluating their inhibitory activity towards acetylcholinesterase.

## 2. Experimental

### 2.1. Chemicals and Reagents

The phenoxy acid herbicides and the transformation products (except 4-chloro-2-methyl-6-nitrophenol that was obtained by synthesis), as well as NaCl, MgCl_2_·6H_2_O, Na_2_HPO_4,_ and NaH_2_PO_4,_ were supplied by Sigma-Aldrich (Sintra, Portugal) and used without further purification. Bovine serum albumin (BSA), 5,5′-dithiobis(2-nitrobenzoic acid) (DTNB), acetylthiocholine iodide (ATCI), galantamine, AChE (CAS 9000-81-1; EC 232-559-3) from electric eel (type VI-s, lyophilized powder), Tris-HCl, and calf thymus DNA, as Type I calf thymus DNA sodium salt (protein content < 3%), were also purchased from Sigma-Aldrich. Ultrapure (Type 1) water (Millipore, Milli Q Gradient) was used throughout the experiments.

The following buffers were used in the acetylcholinesterase activity assay: buffer A: 50 mM Tris-HCl, pH 8; buffer B: 50 mM Tris-HCl, pH 8, containing 0.1% BSA; and buffer C: 50 mM Tris-HCl, pH 8, containing 0.1 M NaCl and 0.02 M MgCl_2_·6H_2_O. Acetylcholinesterase from* Electrophorus electricus *(electric eel) (type VI-s, lyophilized powder, 425 U/mg, 687 mg/protein) was used in the enzymatic assay. The lyophilized enzyme was dissolved in buffer A to make 1000 U/mL stock solution and further diluted with buffer B to get 0.44 U/mL of enzyme in the microplate well.

A stock solution (1.0 × 10^−3^ mol dm^−3^) of DNA was prepared by dissolving an appropriate amount of DNA in 0.05 mol dm^−3^ phosphate buffer solution (pH 7.2; ionic strength adjusted to 0.1 mol dm^−3^ with NaCl). Stock solutions (2.0 × 10^−4^ mol dm^−3^) of herbicides were prepared by dissolution of a suitable quantity in ultrapure water.

The UV absorbance measurements were performed in pH 7.2 phosphate buffer solutions with ionic strength 0.01 mol dm^−3^. Working solutions of DNA (7.0 × 10^−5^ mol dm^−3^), herbicides (5.0 × 10^−5 ^mol dm^−3^ or 1.0 × 10^−4^ mol dm^−3^), and DNA/herbicides were prepared by simple dilution of the appropriate amounts of the stock solutions in phosphate buffer and ultrapure water.

The concentration of DNA solutions, expressed in moles of base pairs, was determined at 20°C by UV spectroscopy at 260 nm, using a molar absorption coefficient *ε*_260_ = 13200 mol^−1^ dm^3^ cm^−1^ [22]. A ratio of absorbance at 260 nm to that at 280 nm (*A*_260_/*A*_280_) greater than 1.8 indicated that DNA was sufficiently pure and free from protein [23]. Control experiments under the same conditions and initial concentrations of herbicides were carried out in parallel for comparison. All the solutions were stored in the refrigerator at 4°C.

### 2.2. Synthesis of 4-Chloro-2-methyl-6-nitrophenol

The synthetic procedure was adapted from the literature with slight modifications [24, 25]. To a solution of 4-chloro-2-methylphenol (0.3 g, 2.2 mmol) in CH_2_Cl_2_ (30 mL), NaNO_2_ (0.3 g, 4.0 mmol), Al(HSO_4_)_3_ (0.4 g, 1.4 mmol), and wet SiO_2_ (50% w/w) (0.44 g) were added. The resulting mixture was stirred and the reaction was completed after 90 min. The crude product was extracted with CH_2_Cl_2_ (2 × 15 mL). The organic phases were combined, washed with water (2 × 15 mL), dried over anhydrous Na_2_SO_4,_ and evaporated under reduced pressure. The nitrated product was purified on a silica gel column using CH_2_Cl_2_/MeOH (9 : 1 and 8 : 2 ratio (v/v)) as eluting system. The fractions containing the desired compound were collected and the solvent evaporated in a rotary evaporator.

^1^H NMR (400 MHz, CH_3_OH): *δ* = 2.30 (3H, s, CH_3_), 7.52 (1H, d, *J* = 2.6 Hz, H (1)), 7.92 (1H, d, *J* = 2.6 Hz, H (2)). MS/EI *m*/*z*: 189 (28), 187 (M^+•^, 100).

### 2.3. Evaluation of Acetylcholinesterase Inhibitory Activity

Acetylcholinesterase activity was determined spectrophotometrically using a 96-well Multiskan Ascent microplate reader (Thermo, Electron Corporation) based on Ellman's method, according to a previously described procedure [26]. In each well, the mixture consisted of 25 *μ*L of 15 mM ATCI in water, 125 *μ*L of 3 mM DTNB in buffer C, 50 *μ*L of buffer B, and 25 *μ*L of 10 mM solution of each compound under study (dissolved in a solution of 10% methanol in buffer A). The absorbance was measured at 405 nm. After this step, 25 *μ*L of AChE (0.44 U/mL) was added to each well and the absorbance was measured again at the same wavelength. The rates of the reactions were calculated by Ascent Software version 2.6 (Thermo Labsystems Oy). The rate of the reaction before adding the enzyme was subtracted from that obtained after enzyme addition in order to correct for spontaneous hydrolysis of substrate. The enzymatic activity was calculated by comparing the rates of the samples with the control (10% methanol in buffer A). Galantamine, a reversible competitive inhibitor of acetylcholinesterase, was used as reference compound [27]. The results are expressed as the mean percentage of inhibition obtained from three separate experiments.

### 2.4. UV Spectroscopy Experiments

The absorption spectra, as well as the UV melting curves, were recorded on a hermetic quartz cell with a 1 cm path length, using an Agilent 8453 UV-Vis spectroscopy system equipped with a thermostatic cell holder. The temperature of the samples was controlled using a Julabo F25/HP thermostatic bath. The UV absorption spectra of herbicides and DNA/herbicides solutions were acquired in the wavelength range of 200–400 nm at 20°C. For DNA melting studies, the temperature of the cell was changed from 20 to 95°C, with a heating rate of 1°C min^−1^.

The fraction of melted base pairs, *θ*, at each temperature, has been calculated from the curves of absorbance versus temperature as described elsewhere [28]. The melting temperature, *T*_*m*_, defined as the temperature at which half of the amount of DNA is denatured, was determined by interpolation in the curves *θ* = *f*(*T*) for *θ* = 0.5 [28]. The hyperchromicity at 260 nm, *H*_260_, was calculated as described elsewhere [28].

### 2.5. Thermodynamic Parameters of DNA Thermal Denaturation

The thermodynamic parameters of DNA thermal denaturation were obtained from the denaturation curves, by two different methods based on the van't Hoff equation [28]. Both methods assume that denaturation is a two-state transition and the values of enthalpy and entropy are not dependent on temperature.

Briefly, the first method is based on the dependence of the equilibrium constant, *K*, of DNA denaturation on temperature, *T*. The value of *K* at each temperature can be expressed as a function of the broken base pairs fraction, *θ*. The van't Hoff denaturation enthalpy and entropy values were obtained by linear regression of −ln⁡*K* versus 1/*T* and fitting the data to values of *θ* from 0.25 to 0.75 [28]. In the second method, the van't Hoff denaturation enthalpy and entropy values were calculated using the peak height maxima obtained from the derivative melting curves, (*dθ*/*dT*)_max⁡_ [28].

Values of Δ*H*_vH_^o^ and Δ*S*_vH_^o^ obtained from both methods differed by less than ±1%. Thus, the values given correspond to the average calculated from the values obtained using both methods.

## 3. Results and Discussion

### 3.1. Evaluation of Acetylcholinesterase Inhibitory Activity

The inhibition of peripheral enzymes, such as acetylcholinesterase, provides a convenient and nondestructive mean of monitoring exposure to pesticides and their transformation products and has been widely implemented by regulatory agencies [29]. Monitoring of AChE inhibition is widely used as a biomarker of organophosphorus and carbamate pesticide exposure either in aquatic or terrestrial environments [29]. A number of important contaminants, other than these pesticide groups, have recently been shown to have anticholinesterase properties, namely, heavy metals and herbicides [29].

The* in vitro* screening assay most frequently used to evaluate the inhibitory activity of a contaminant towards AChE is based on Ellman's method [30]. Accordingly, the chlorophenoxy herbicides and their transformation products were evaluated for their inhibitory activities toward AChE, in comparison with galantamine as reference drug. The anticholinesterase activities are summarized in Table 1. No significant inhibitory activities towards AchE were found for the chlorophenoxy herbicides under study, when compared with the reference drug. However, the results found revealed that the transformation products displayed a rather noticeable inhibitory activity when compared with the parent herbicides. The data obtained show that the nitroderivatives exhibit the highest inhibitory activity among all the tested compounds. Actually, the inhibitory activity of the compounds depends on the type and number of substituents attached to the aromatic ring. These results reinforce the idea that a particular attention should be placed on assessing the effect of neurotoxicity of pesticides, as, in some cases, these effects may only be observed after the formation of transformation products, which arise from environmental degradation.

### 3.2. Evaluation of Pesticides-DNA Interactions

DNA is found in cells and usually looks like a right-handed double helix. The two chains (strands) of the double helix are connected by hydrogen bonds. Small ligand molecules can bind to DNA and artificially alter and/or inhibit the functioning of DNA. In general, four types of reversible binding modes can occur between molecules and double-helical DNA: (i) electrostatic attractions with the anionic sugar-phosphate backbone of DNA, (ii) interactions with the major and minor grooves, (iii) intercalation between base pairs via the DNA major and minor grooves, and (iv) a threading intercalation mode [14]. Depending on structural features of both the molecule and DNA, some molecules can present simultaneously more than a single interaction mode with DNA [14].

The mechanism of interactions between small molecules and DNA is still unclear. Thus, various techniques have been used to study the binding of small molecules with DNA with UV-visible absorption spectroscopy being the simplest and most commonly employed instrumental technique.

#### 3.2.1. UV Absorption Spectra

Some pesticides and/or its environmental transformation products have the capacity to interact and damage the structure of the DNA, a process that often has a toxic outcome for human health. Thus, the determination of the specific type of structural DNA damage can be viewed as a biomarker of damage exposure [31]. The pesticide-DNA interaction can be efficiently detected by UV-Vis absorption spectroscopy by measuring the changes in the absorption properties of the pesticide or the DNA [32, 33]. The magnitude of the shift of the position of the absorption bands could be interpreted as an indication of the strength of the DNA-pesticide interaction [33].

**Figure 2:**
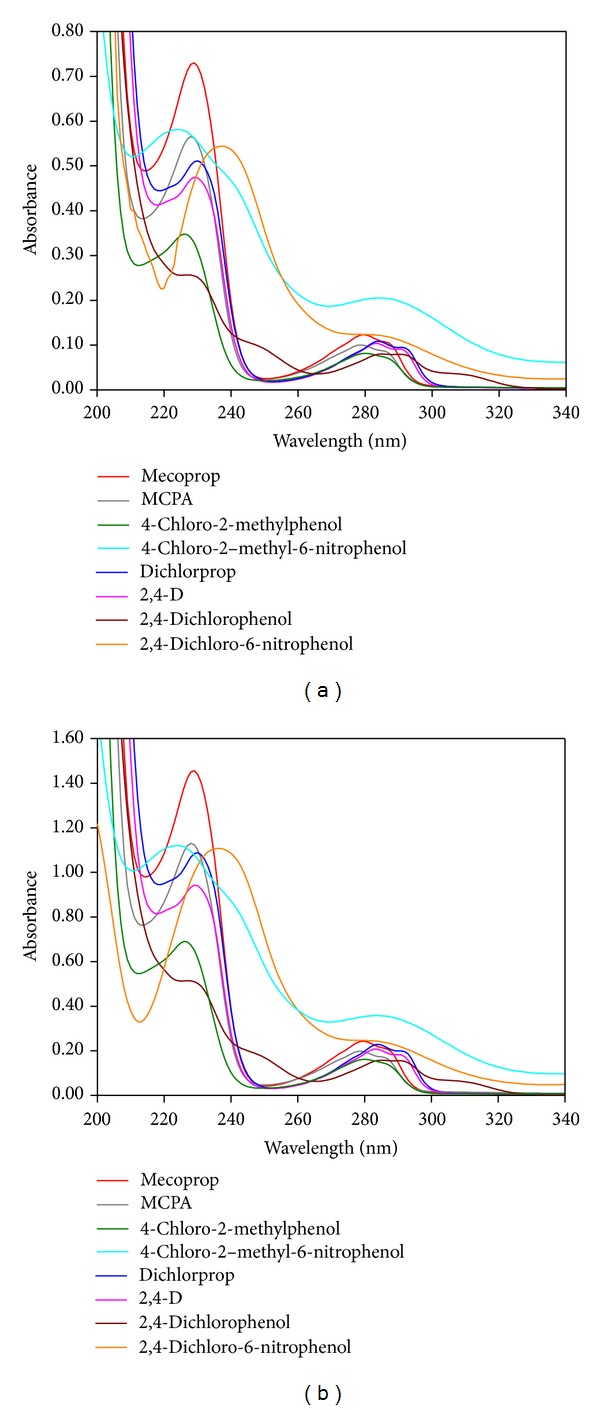
UV-Vis absorption spectra of (a) 5.0 × 10^−5 ^mol dm^−3^ and (b) 1.0 × 10^−4 ^mol dm^−3^ of chlorophenoxy herbicides and TPs standard solutions in pH 7.2 phosphate buffer electrolyte.

The UV absorption spectra of chlorophenoxy herbicides, at two different concentrations, in the absence of DNA are presented in Figure 2. Except for the nitroderivatives, where only two bands are seen, all the other studied compounds exhibit three absorption bands. The weaker bands, in the 270–300 nm range, correspond to *π* → π* and *n* → π* transitions that are characteristic of benzene and its substituted derivatives; the band corresponding to the maximum absorption, occurring below 240 nm, is also related to the existence of an aromatic system [34, 35]. When nonbonding pair substituents (such NO_2_) are present on the benzene ring, the absorptions are shifted substantially to longer wavelengths and the fine structure of the B-band is seriously diminished or wholly eliminated, because of *n*-*π* conjugation [34]. These changes can justify the occurrence of a single band in the 270–300 nm range for nitroderivatives.

**Figure 3:**
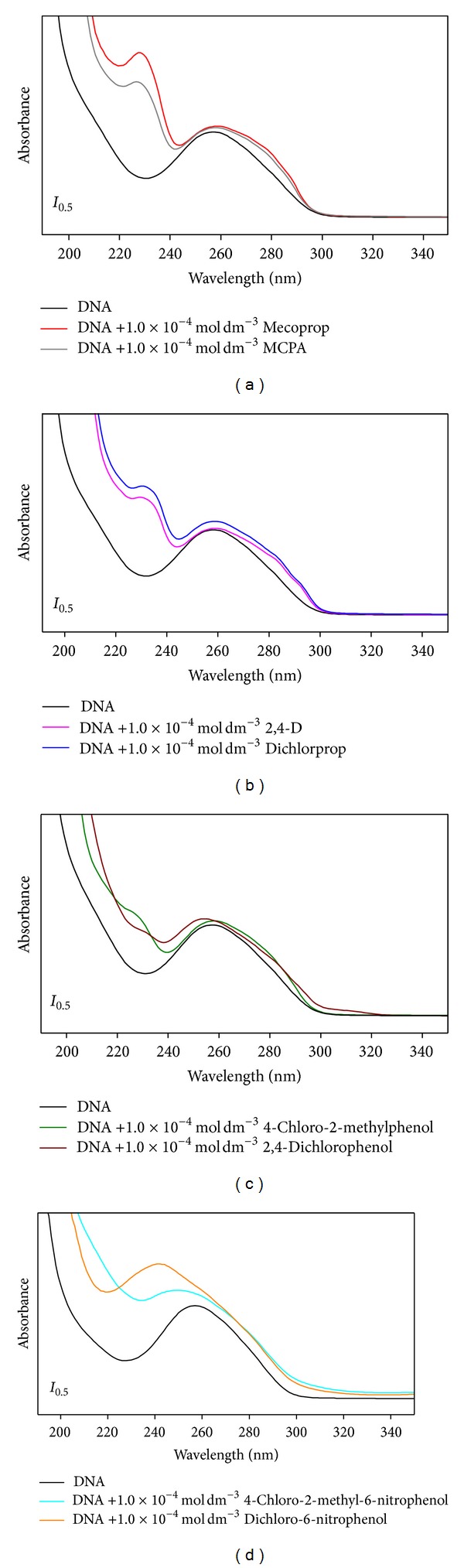
UV-Vis absorption spectra of 7.7 × 10^−5^ mol dm^−3^ of DNA in the absence and presence of 1.0 × 10^−4^ mol dm^−3^ of (a) mecoprop and MCPA, (b) 2,4-D and dichlorprop, (c) 4-chloro-2-methylphenol and 2,4-dichlorophenol, and (d) 4-chloro-2-methyl-6-nitrophenol and 2,4-dichloro-6-nitrophenol in pH 7.2 phosphate buffer electrolyte.

Figure 3 shows the UV-visible absorption spectra obtained for DNA in the absence or presence of chlorophenoxy herbicides and their transformation products (DNA/herbicides and DNA/TPs). The UV-Vis absorption spectrum of DNA exhibits a broad band (200–340 nm) in the UV region with a maximum absorption at 260 nm (Figure 3). This maximum is a consequence of the chromophoric groups in purine and pyrimidine moieties responsible for the electronic transitions [32, 33]. Spectra registered for DNA/herbicides and DNA/TPs solutions exhibit the absorption bands common to both DNA and each of the herbicides or TPs under study (Figure 3). Except for the nitroderivatives, for both concentrations tested only slight variations are observed in the peak position and absorption intensity of the bands for DNA/herbicides solutions compared to DNA.

**Figure 4:**
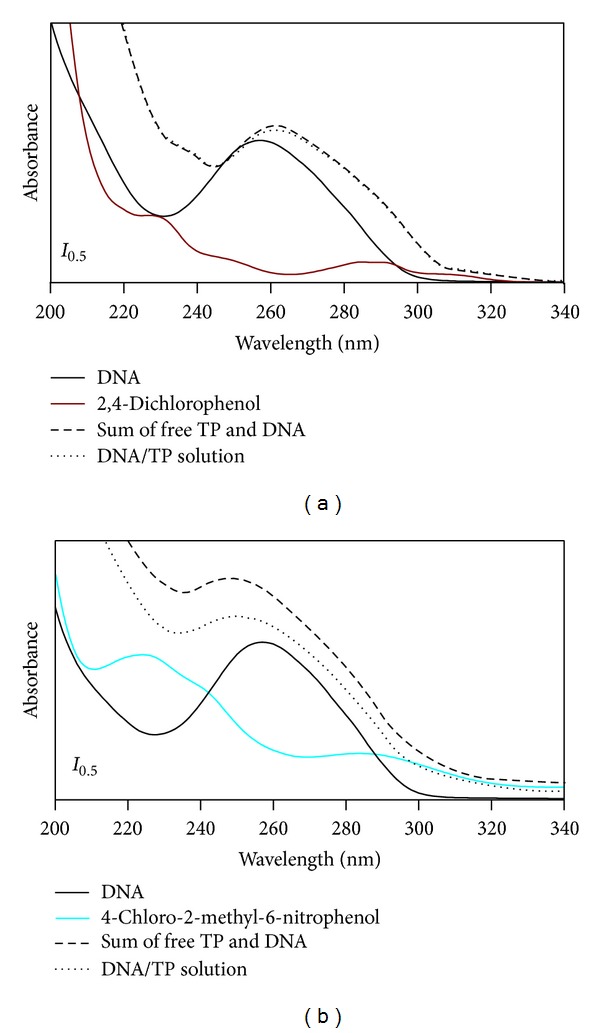
UV-Vis absorption spectra of 7.7 × 10^−5 ^mol dm^−3^ of DNA in the absence and presence of 1.0 × 10^−4 ^mol dm^−3^ of (a) 2,4-dichlorophenol and (b) 4-chloro-2-methyl-6-nitrophenol in pH 7.2 phosphate buffer electrolyte.

To evaluate the effect of chlorophenoxy herbicides and transformation products on the UV spectrum of DNA, the spectra of DNA/herbicides and DNA/TPs solutions were compared with the sum of the individual spectra of DNA and herbicide or TP, at the same concentration (Figure 4). Except for the nitroderivatives, the UV spectra of DNA/herbicides and DNA/TPs solutions present only minor changes in the absorption intensity of the bands relative to the sum of the spectra of DNA and each of the herbicides and TPs (Figure 4(a)). Since no significant variations are observed, one can assume that the interaction of these herbicides and TPs with DNA base pairs is not strong, since no marked effect was observed in the structure of the DNA molecule. On the contrary, for the nitroderivatives (2,4-dichloro-6-nitrophenol and 4-chloro-2-methyl-6-nitrophenol) (Figure 4(b)), a meaningful bathochromic shift (7–10 nm) and a decrease in intensity (hypochromism) are observed for the band at ~260 nm of DNA which might indicate that both these compounds interact with DNA [33]. The shifts observed in absorbance and wavelength of DNA characteristic band can reflect the structural changes of DNA, namely, in the stacking pattern, disruption of the hydrogen bonds between complementary strands, covalent binding to the DNA bases, and groove binding or intercalation of aromatic rings of molecules between adjacent base pairs [33, 36].

#### 3.2.2. DNA Melting Studies

The two strands of DNA are held together mainly by stacking interactions, hydrogen bonds, and hydrophobic effects between the complementary bases. When a DNA solution is heated, the double helix separates into two single strands in a process known as DNA denaturation, and the temperature at which the DNA strands are half-denatured is called the melting temperature, *T*_*m*_ [32]. During the disruption of the double helical structure, the base-base interactions will be reduced, increasing the UV absorbance of DNA solution because many bases are in free form and do not form hydrogen bonds with complementary bases [33]. A shift in *T*_*m*_, to values different from native ds-DNA, is an indication that a drug-DNA interaction exists. The magnitude of the shift depends on the type of interaction. Thus, for intercalating agents, the increase observed in the *T*_*m*_ value is higher than in the case of agents interacting through the DNA minor or major grooves [32]. Thus, UV-Vis spectroscopy becomes a very valuable technique to determine the melting temperatures and to study the interaction of small molecules with DNA.

**Figure 5:**
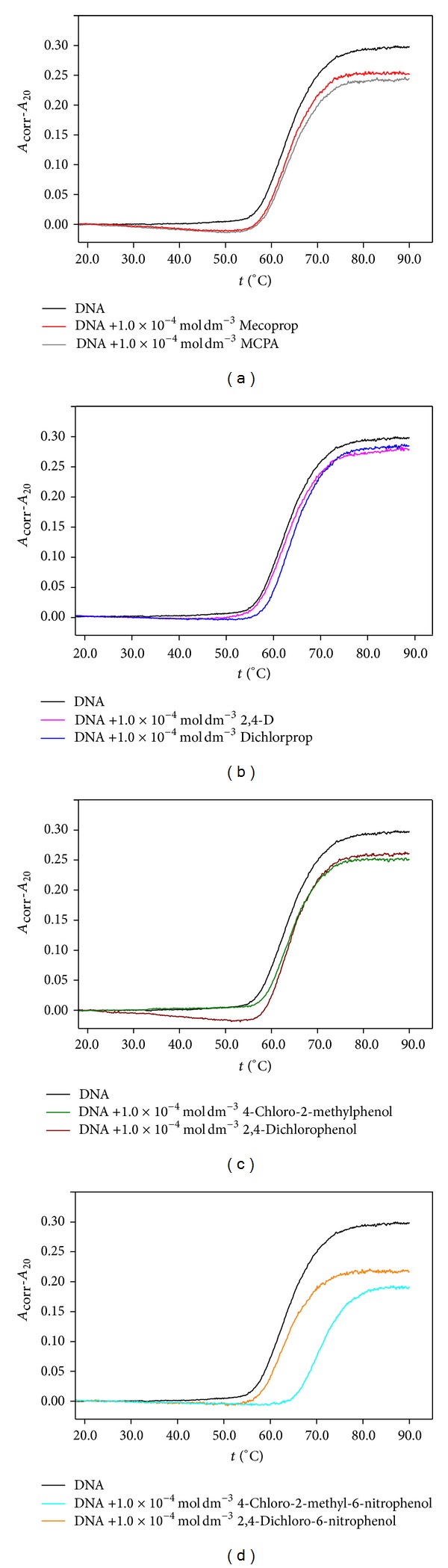
Corrected absorbance at 260 nm versus temperature for 7.7 × 10^−5 ^mol dm^−3^ of DNA in the absence and presence of 1.0 × 10^−4 ^mol dm^−3^ of (a) mecoprop and MCPA, (b) 2,4-D and dichlorprop, (c) 4-chloro-2-methylphenol and 2,4-dichlorophenol, and (d) 4-chloro-2-methyl-6-nitrophenol and 2,4-dichloro-6-nitrophenol in pH 7.2 phosphate buffer electrolyte.

The DNA melting curves at 260 nm for DNA, DNA/herbicides, and DNA/TPs solutions are shown in Figure 5. As the absorbance obtained for the herbicides and TPs at 260 nm depends on temperature, all denaturation curves were corrected to this effect by subtracting from the melting curves of DNA/herbicides and DNA/TPs solutions the values of absorbance corresponding to the solutions containing only herbicides or TPs. Therefore, the resulting curves (Figure 5) reflect only the change in absorbance due to temperature increase resulting from DNA denaturation. The data found allow concluding that all compounds under study reduce the hyperchromism along the DNA denaturation process. In fact, above 80°C (temperature at which it is assumed that the strands of DNA have been totally separated), there is a reduction in hyperchromism as a result of the presence of herbicides and TPs under study, with this effect being more noticeable for the nitroderivatives (Figure 5). Moreover, the hyperchromism observed is influenced by the herbicide or TP concentration: an increase of concentration leads to a decrease of the DNA absorbance upon denaturation.

**Figure 6:**
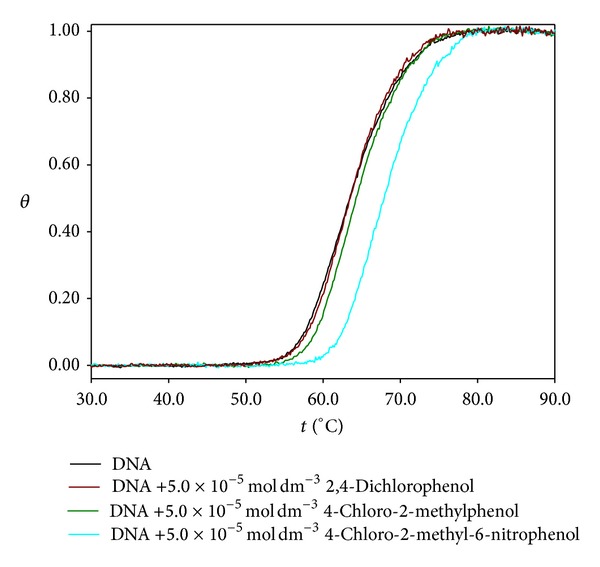
Molar fraction of denatured DNA versus temperature for 7.7 × 10^−5^ mol dm^−3^ of DNA in the absence and presence of 5.0 × 10^−5^ mol dm^−3^ of TPs in pH 7.2 phosphate buffer electrolyte.

The values of hyperchromism, *H*_260_, calculated at 85°C, are presented in Table 2. The reduction of the hyperchromicity observed in the DNA melting curves indicates that an interaction of the studied herbicides and their TPs with DNA occurs. In general, the interactions which occur between DNA and chlorophenoxy herbicides as well as their chlorophenol transformation products are weak. However, the insertion of a nitro substituent on the aromatic ring (nitroderivatives) seems to induce stronger interactions. The curves of molar fraction of denatured DNA versus temperature (Figure 6) show that the denaturation temperature values are not significantly affected by herbicides and TPs, except for 4-chloro-2-methyl-6-nitrophenol (Table 2).

Spectroscopy was used to assess the thermal stability of the DNA in comparison with the DNA/herbicide and DNA/TPs solutions. From the calculated thermodynamic parameters, it is possible to infer that the presence of the herbicides and TPs causes a slight increase in entropy and enthalpy of DNA denaturation (Table 2). In fact, although the denaturation temperature values are not significantly different (with the exception of 4-chloro-2-methyl-6-nitrophenol), the denaturation enthalpy values for DNA/herbicides solutions are generally higher than the observed for DNA. These results suggest that a stabilization of the DNA double helix may occur in the presence of herbicides, although no influence on the relative stability of base pairs is observed. External binding to the major or minor DNA grooves can be responsible for the observed disturbance on DNA conformation [32, 33]. For the transformation product 4-chloro-2-methyl-6-nitrophenol, a noteworthy increase in *T*_*m*_ can be observed. This is a clear indication of a strong interaction with the DNA molecule, increasing its stability.

The differing behaviour of 4-chloro-2-methyl-6-nitrophenol compared to all other herbicides and chlorophenols under study is in accordance with data found in the literature. In fact, the interactions of nitroaromatic compounds with DNA, and the resulting mutagenic properties, have been characterized for a variety of monocyclic, polycyclic, and heterocyclic nitroaromatic compounds [37]. However, one can remark the different behaviour observed for 4-chloro-2-methyl-6-nitrophenol relative to 2,4-dichloro-6-nitrophenol. The observed differences can be explained by the position, and inherent substituent effect, of the group found into 2-position of the aromatic ring. Several structural factors can contribute for the toxicity of chloronitrophenols, namely, the presence of (1) an acid-dissociable group (hydroxyl substituent), (2) strong-withdrawing moieties (halogen and nitrogroups), and (3) a bulky hydrophobic group (a benzene ring). The hydroxyl group decreases hydrophobicity but increases reactivity and the addition of chloro- and nitrogroups increases both hydrophobicity and the acidic strength (reactivity) of phenol. The strength of the toxic effect also stems from localization of the substituent in the aromatic nucleus. Actually, it is described that the position of the chlorine substituents on the phenol ring is extremely important for the observed toxicity [38]. The existence of a chlorosubstituent in the* ortho *position in phenol molecule usually decreases its toxicity [38]. Accordingly, it can be one rationale to explain the toxicity outline observed for the two chloronitrophenols under study.

## 4. Conclusion

The consequences of pesticide use on natural ecosystems represent an important issue in the field of environmental chemistry. Recently, it has been shown that the formation of by-products in the environment can play a significant role in defining the impact of pesticides on human health [3]. Actually, TPs can be more toxic than the parent molecules, and, consequently, they can represent a greater risk to human health and environment.

The molecular mechanisms related to the toxic effect of chlorophenoxy herbicides and their main TPs have not been completely elucidated. Thus, in this study, the evaluation of the interaction of four chlorophenoxy herbicides as well as their main TPs with calf thymus DNA by UV-visible absorption spectroscopy has been accomplished. Furthermore, the toxicity of the chlorophenoxy herbicides and TPs was also assessed evaluating their inhibitory activity towards acetylcholinesterase.

On the basis of the data obtained, it seems that AChE is not the main target of chlorophenoxy herbicides and their TPs. Nevertheless, the results found revealed that the transformation products displayed a rather noticeable inhibitory activity when compared with the parent herbicides.

The results obtained in the DNA-drug interaction studies showed, for the large majority of the compounds tested, a slight effect on the stability of DNA double helix. Yet, the experimental data demonstrate that a transformation product (4-chloro-2-methyl-6-nitrophenol) interacts with DNA through a noncovalent mode. Furthermore, the results also indicate that strength of the interaction is related to the type and localization of the substituents in the aromatic moiety.

In the future, attention should be given to the risks originating from TPs, since they can pose a higher hazard than their parent pesticides in respect to persistence, bioaccumulation, and toxicity.

## Figures and Tables

**Table 1:** Acetylcholinesterase inhibitory activity of chlorophenoxy herbicides and its transformation products.

Compound	% inhibition ± S.D.^a^
MCPA	12.9 ± 1.5
Mecoprop	Inactive
4-Chloro-2-methylphenol	19.0 ± 0.7
4-Chloro-2-methyl-6-nitrophenol	28.4 ± 5.9
2,4-D	3.9 ± 0.03
Dichlorprop	Inactive
2,4-Dichlorophenol	22.9 ± 1.6
2,4-Dichloro-6-nitrophenol	35.8 ± 4.2
Galantamine	95.7 ± 0.05

^a^Results are expressed as mean ± standard deviation of three independent determinations.

**Table 2:** Thermodynamic parameters of DNA thermal denaturation, obtained by UV spectroscopy, in solutions with different herbicides^a,b^.

Compound	*t*_*m*_/°C	−Δ(*H*_260_ (85°C))	Δ(Δ*H*_vH_°)/kJ mol^−1^	Δ(Δ*S*_vH_°)/kJ K^−1^ mol^−1^
MCPA	63.4 ± 0.6	0.06	21 ± 8	0.06 ± 0.02
	63.9 ± 0.4	0.07	0	0

Mecoprop	63.7 ± 0.6	0.03	7 ± 7	0.02 ± 0.02
	63.7 ± 0.6	0.06	31 ± 8	0.09 ± 0.02

2,4-D	63.0 ± 0.3	0	14 ± 6	0.04 ± 0.01
	63.2 ± 0.6	0.03	9 ± 8	0.03 ± 0.02

Dichlorprop	64.1 ± 0.5	0.03	46 ± 7	0.13 ± 0.01
	64.7 ± 0.5	0.05	29 ± 7	0.08 ± 0.01

2,4-Dichlorophenol	64.3 ± 0.5	0.07	15 ± 6	0.04 ± 0.01
	64.0 ± 0.5	0.09	33 ± 6	0.09 ± 0.01

4-Chloro-2-methylphenol	64.3 ± 0.3	0	15 ± 5	0.04 ± 0.01
	64.3 ± 0.4	0.05	30 ± 6	0.08 ± 0.01

2,4-Dichloro-6-nitrophenol	63.5 ± 0.5	0.05	0	0.08 ± 0.01
	63.6 ± 0.4	0.05	4 ± 6	0.08 ± 0.01

4-Chloro-2-methyl-6-nitrophenol	67.9 ± 0.5	0.05	0	0.08 ± 0.01
	70.8 ± 0.5	1	42 ± 7	0.08 ± 0.02

^a^[DNA] = 7.7 × 10^−5^ mol dm^−3^, phosphate buffer solution, ionic strength = 0.01 mol dm^−3^. For each herbicide or TP, the top values correspond to a concentration of 5.1 × 10^−5^ mol dm^−3^ and the bottom values to 1.0 × 10^−4^ mol dm^−3^.

^b^Δ(*H*_260_ (85°C)) = *H*_260_  (85°C)_(DNA + herbicide or TP)_ − *H*_260_  (85°C)_(DNA)_; Δ(Δ*H*_vH_°) = Δ*H*_vH (DNA + herbicide or TP)_° − Δ*H*_vH (DNA)_°; Δ(Δ*S*_vH_°) = Δ*S*_vH (DNA + herbicide or TP)_° − Δ*S*_vH (DNA)_°.

## References

[1] FAO (2009). *Global Agriculture Towards 2050*.

[2] Manahan SE (2005). *Environmental Chemistry*.

[3] Escher BI, Fenner K (2011). Recent advances in environmental risk assessment of transformation products. *Environmental Science and Technology*.

[4] Alavanja MCR, Hoppin JA, Kamel F (2004). Health effects of chronic pesticide exposure: cancer and neurotoxicity. *Annual Review of Public Health*.

[5] Perry MJ (2008). Effects of environmental and occupational pesticide exposure on human sperm: a systematic review. *Human Reproduction Update*.

[6] Andreu V, Picó Y (2004). Determination of pesticides and their degradation products in soil: critical review and comparison of methods. *TrAC—Trends in Analytical Chemistry*.

[7] Cheung J, Rudolph MJ, Burshteyn F (2012). Structures of human acetylcholinesterase in complex with pharmacologically important ligands. *Journal of Medicinal Chemistry*.

[8] Kamel F, Hoppin JA (2004). Association of pesticide exposure with neurologic dysfunction and disease. *Environmental Health Perspectives*.

[9] Casida JE (2009). Pest toxicology: the primary mechanisms of pesticide action. *Chemical Research in Toxicology*.

[10] JanakiDevi V, Nagarani N, YokeshBabu M, Kumaraguru AK, Ramakritinan CM (2013). A study of proteotoxicity and genotoxicity induced by the pesticide and fungicide on marine invertebrate (Donax faba). *Chemosphere*.

[11] Fan JY, Geng JJ, Ren HQ, Wang XR, Han C (2013). Herbicide Roundup and its main constituents cause oxidative stress and inhibit acetylcholinesterase in liver of Carassius auratus. *Journal of Environmental Science and Health B*.

[12] Chaires JB (1998). Drug-DNA interactions. *Current Opinion in Structural Biology*.

[13] Simoniello MF, Kleinsorge EC, Scagnetti JA, Grigolato RA, Poletta GL, Carballo MA (2008). DNA damage in workers occupationally exposed to pesticide mixtures. *Journal of Applied Toxicology*.

[14] Ahmadi F, Stoytcheva M (2011). The impacts of pesticides exposure. *Pesticides*.

[15] Eurostat (2007). *The Use of Plant Protection Products in the European Union: Data 1992–2003*.

[16] Kiely T, Donaldson D, Grube A (2004). *Pesticides Industry Sales and Usage—2000 and 2001 Market Estimates*.

[17] Jankowska A, Biesaga M, Drzewicz P, Trojanowicz M, Pyrzyńska K (2004). Chromatographic separation of chlorophenoxy acid herbicides and their radiolytic degradation products in water samples. *Water Research*.

[18] (1987). Chlorophenoxy herbicides. *International Agency for Research on Cancer*.

[19] Chiron S, Minero C, Vione D (2007). Occurrence of 2,4-dichlorophenol and of 2,4-dichloro-6-nitrophenol in the Rhône river delta (Southern France). *Environmental Science and Technology*.

[20] Chiron S, Comoretto L, Rinaldi E, Maurino V, Minero C, Vione D (2009). Pesticide by-products in the Rhône delta (Southern France). The case of 4-chloro-2-methylphenol and of its nitroderivative. *Chemosphere*.

[21] Heng ZC, Ong T, Nath J (1996). In vitro studies on the genotoxicity of 2,4-dichloro-6-nitrophenol ammonium (DCNPA) and its major metabolite. *Mutation Research—Genetic Toxicology*.

[22] Reichmann ME, Rice SA, Thomas CA, Doty P (1954). A further examination of the molecular weight and size of desoxypentose nucleic acid. *Journal of the American Chemical Society*.

[23] Marmur J (1961). A procedure for the isolation of deoxyribonucleic acid from micro-organisms. *Journal of Molecular Biology*.

[24] Habibi D, Zolfigol MA, Shiri M, Sedaghat A (2006). Nitration of substituted phenols by different efficient heterogeneous systems. *South African Journal of Chemistry*.

[25] Ramachandran K, Sivakumar P, Suganya T, Renganathan S (2011). Production of biodiesel from mixed waste vegetable oil using an aluminium hydrogen sulphate as a heterogeneous acid catalyst. *Bioresource Technology*.

[26] Pereira DM, Ferreres F, Oliveira J, Valentão P, Andrade PB, Sottomayor M (2009). Targeted metabolite analysis of Catharanthus roseus and its biological potential. *Food and Chemical Toxicology*.

[27] Sramek JJ, Frackiewicz EJ, Cutler NR (2000). Review of the acetylcholinesterase inhibitor galanthamine. *Expert Opinion on Investigational Drugs*.

[28] Benfeito S, Garrido J, Sottomayor MJ, Borges F, Garrido EM, Kobayashi D, Watanabe E (2013). Pesticides and cancer: studies on the interaction of phenoxy acid herbicides with DNA. *Handbook on Herbicides: Biological Activity, Classification and Health & Environmental Implications*.

[29] Lionetto MG, Caricato R, Calisi A, Schettino T, Visser JE (2011). Acetylcholinesterase inhibition as a relevant biomarker in environmental biomonitoring: new insights and perspectives. *Ecotoxicology Around the Globe*.

[30] Ellman GL, Courtney KD, Andres V, Featherstone RM (1961). A new and rapid colorimetric determination of acetylcholinesterase activity. *Biochemical Pharmacology*.

[31] Shugart LR (2000). DNA damage as a biomarker of exposure. *Ecotoxicology*.

[32] González-Ruiz V, Olives AI, Martín MA, Ribelles P, Ramos MT, Menéndez JC, Olsztynska S (2011). An overview of analytical techniques employed to evidence drug-DNA interactions. *Applications to the Design of Genosensors in Biomedical Engineering, Trends, Research and Technologies*.

[33] Sirajuddin M, Ali S, Badshah A (2013). Drug-DNA interactions and their study by UV-Visible, fluorescence spectroscopies and cyclic voltametry. *Journal of Photochemistry and Photobiology B*.

[34] Kalsi PS (2004). *Spectroscopy of Organic Compounds*.

[35] Pavia DL, Lampman GM, Kriz GS, Vyvyan JR (2009). *Introduction to Spectroscopy*.

[36] Suh D, Chaires JB (1995). Criteria for the mode of binding of DNA binding agents. *Bioorganic and Medicinal Chemistry*.

[37] Purohit V, Basu AK (2000). Mutagenicity of nitroaromatic compounds. *Chemical Research in Toxicology*.

[38] Pouretedal HR, Keshavarz MH (2011). Prediction of toxicity of nitroaromatic compounds through their molecular structures. *Journal of the Iranian Chemical Society*.

